# Suicide and sociodemographic factors among older adults in Norway: a register-based study

**DOI:** 10.1007/s00127-025-03007-x

**Published:** 2025-10-21

**Authors:** Anna Torp Johansen, Sissel Marguerite Bélanger, Anne Reneflot, Erlend Hem, Eivind Aakhus, Carine Øien-Ødegaard, Kim Stene-Larsen, Cecilie Bhandari Hartberg

**Affiliations:** 1 Department of Old Age Psychiatry, Division of Mental Health and Addiction, Oslo University Hospital, Oslo, Norway; 2 Department of Behavioral Science in Medicine, Institute of Basic Medical Sciences, Faculty of Medicine, University of Oslo, Oslo, Norway; 3 Department of Mental Health, Norwegian Institute of Public Health, Oslo, Norway; 4 Institute for Studies of the Medical Profession, Oslo, Norway; 5 National Norwegian Centre for Ageing and Health, Vestfold Hospital Trust, Tønsberg, Norway; 6 Division of Mental Health and Addiction, Oslo University Hospital, Oslo, Norway

**Keywords:** Suicide, Older adults, Sociodemographic factors, Sex characteristics, Age categories

## Abstract

**Purpose:**

Suicide rates are high among older adults, yet research on risk factors associated with suicide in this age group remain largely unexplored. This study aims to examine the relationship between suicide and sociodemographic characteristics among individuals aged 60 and older.

**Methods:**

We utilized data from Norwegian national registries, identifying individuals aged 60 and older who died by suicide between 2005 and 2019 (*n* = 2060, 70.7% males), and used a case-control design. To examine the relationships between suicide and sociodemographic factors, we used descriptive analyses and conditional multivariate logistic regression analyses, stratified by sex and age categories.

**Results:**

Among individuals aged 60 to 69, risk factors for suicide included receiving a disability pension (odds ratio (OR) = 2.79 males, OR = 7.71 females), having mixed income sources (OR = 1.79 males, OR = 3.70 females), living alone (OR = 2.49 males, OR = 2.46 females), and living in urban areas, which was associated with an increased risk for females (OR = 1.85). Among males, living alone was also a significant risk factor for suicide in the 70 to 79 age group (OR = 1.85), and those aged 80 and above (OR = 2.16). Living in rural areas reduced risk for females aged 80 and above (OR = 0.05).

**Conclusion:**

This first register-based study of suicide in older adults in Norway highlights that living arrangements, urbanization level, and income source are significant risk factors for suicide among older adults. Interventions to improve social connectedness, with a focus on the urban-rural divide, could potentially reduce suicide risk. Sex and age categories should be considered in future research and when implementing preventive measures.

**Supplementary Information:**

The online version contains supplementary material available at 10.1007/s00127-025-03007-x.

## Background

Populations across the world are shifting rapidly towards older age, leading to increased proportions of older adults and increased life expectancies in general, which in turn will place greater demands on healthcare and social support systems. Suicide is one of the leading causes of premature mortality worldwide and remains a critical public health issue. The results of previous research have shown that, among elderly men, suicide rates are higher than age-standardized rates in most countries [1]. Among females, the pattern is less clear [1]. In Norway, the suicide rate across all age groups in the period 2019 to 2023 was 17.3/100,000 for males and 6.8/100,000 for females. For people aged 75 and older, suicide rates were 24.3/100,000 for males and 5.6/100,000 for females [2]. Despite the fact that the rates are highest among the oldest males, suicide among older adults remain largely unexplored in Norway, as well as in most high-income countries. Older adults have received less research attention, yet with an aging population the number of suicides among older adults is expected to increase, and it is crucial to address the current knowledge gaps.

Previous results from studies on older adults have shown that increased suicide risk is associated with lower educational levels and living alone in males [3–5]; urban residence in females [5, 6]; separation, divorce, and widowhood [7]; lower income levels [8]; receiving disability pensions compared to receiving retirement pensions [6]; and involuntary retirement [9] across both sexes. Some factors may reduce risk and serve as protective factors, such as having children and grandchildren [10, 11]. Even though several studies explore associations between social factors and suicide risk among older adults, few studies have focused on death by suicide as the primary outcome or used high-quality nationwide register data. In addition, few of the studies addressing risk factors are from recent years. Since risk factors may vary across countries [12], there is a need for country-specific research, and social-risk factors in old age have not yet been systematically investigated in Norway. An updated and more comprehensive understanding of the sociodemographic characteristics of older adults who have died by suicide is essential for informing health authorities. This knowledge will aid in developing preventive measures for older adults in general, and specifically for those living in Norway.

Older adults are not a homogenous group. The period of old age can span two or more decades, encompassing multiple life phases, from the early old age to the oldest old age. Each phase is associated with different life circumstances. Given that suicide risk varies across different age groups of older adults and between sexes, it is likely that different risk factors are at play across these categories.

The aim of this study is to examine the sociodemographic risk and protective factors of individuals aged 60 and older who died by suicide in Norway between 2005 and 2019. This will be achieved by assessing how variations in sociodemographic characteristics differ between the group who died by suicide and a representative same-age control group from the general population. Given the differences in rates, we investigated the age and sex categories separately. By using high-quality nationwide registry data, we are able to conduct comprehensive analyses of a broad range of relevant sociodemographic factors, allowing for a more detailed understanding of suicide risk and protective factors in later life.

## Materials and methods

### Data sources

Using four Norwegian nationwide administrative registries, we linked data with unique identification codes to create individual record linkages. The primary outcome variable, death by suicide, was extracted from the Norwegian Cause of Death Registry. Sociodemographic variables were extracted from the National Educational Database, the Norwegian National Population Registry, and the Norwegian Social Security Database. The registries are updated annually, and registration is mandatory for the entire population.

## Study population

The study population included individuals aged 60 and older who died by suicide, as identified in the Norwegian Cause of Death Registry (ICD-10 codes X60-X84, Y87.0) between 2005 and 2019. To ensure that only individuals residing in Norway at the time of death were included, those with a registration status of either “emigrated” or “unregistered” in the Norwegian National Population Registry were excluded. To ensure that we included cases with complete observations, individuals with missing data in one or more independent variables were excluded. Among the 2,114 suicides registered during 2005–2019, 54 (2.5%) were excluded in total. Of these, 39 (1.8%) due to missing information on independent variables and 15 (0,7%) due to registration status. To assess potential biases introduced by these exclusions, we compared the included cases with excluded individuals on age, sex, and immigration status. No significant differences were found for age. Significant differences were observed for immigration status (χ²=19.6, *p* < 0.001): 6 of the 15 individuals excluded due to registration status had an immigration background. Significant differences were observed for sex (χ²=8.4, *p* = 0.004), primarily explained by the exclusion of 24 individuals with negative values for registered working income, of whom 22 were males. A flow chart of the exclusion process is provided in supplementary Figure S1.

For the present study we used a case–control design. Although register data would have permitted a cohort study, our objective was not to estimate incidence or time-to-event outcomes but to examine associations between sociodemographic factors and suicide in older adults. Given the rarity of suicide, a case–control design offers an efficient and appropriate framework for addressing our aim. Each suicide case was matched with five controls from the general population who were alive in the index year (i.e., the year of the case’s death), with matching based on sex and age. Controls were selected using cumulative sampling from a person-year file, where each individual contributed one observation for every year they were alive during the observation period. Individuals who died by suicide were excluded from this pool of data. From the eligible data set, random sampling was applied to select five controls for each case, and each individual could serve as a control only once. For time-varying variables, measures were obtained at the same time-points for cases and controls.

## Variables

All variables were extracted from the calendar year in which the suicide occurred, unless otherwise specified. The age threshold was set at 60 years of age and older, in accordance with the United Nations’ definition of older adults [13]. In Norway, a significant proportion of individuals retire or transition into retirement in their early sixties, making this an appropriate threshold to investigate specific factors pertaining to late-life suicide risk. Birth year was extracted from the Norwegian National Population Registry and used to calculate age, which was further divided into three age categories: 60 to 69, 70 to 79, and 80 or older.

Sex was obtained from the Norwegian National Population Registry and categorized as male or female, consistent with the registry’s classification. Education level was measured in the case individual’s year of death, which was derived from National Educational Database and categorized into three levels. No/primary education includes individuals with no formal education or only elementary-level education, secondary education includes individuals with high school-level education, and higher education includes individuals with post-secondary education and university-level qualifications. The migration status variable was derived from the Norwegian National Population Registry and categorized as a binary variable. Individuals born abroad with one or two foreign-born parents and individuals born in Norway with two foreign-born parents were classified as migrants. The remaining sample, including Norwegian-born individuals with Norwegian parents or foreign-born individuals with Norwegian parents, was categorized as the majority population.

Urbanization levels were determined using registered municipality of residence from The Norwegian Population Registry Statistics Norway’s centrality index [14]. This index, originally divided into six levels reflecting travel time to workplaces and services, was grouped into three broader categories: rural (the least central areas), sub-rural (intermediate levels of centrality), and urban (the most central areas).

Marital status was obtained from the Norwegian National Population Registry. The categories used include never married, married, widowed, and a combined category for separated and divorced.

The Norwegian National Population Registry contains links between parents and offspring, which were used to indicate whether they did or did not have offspring. The variable family status was created with three defined categories: having children, having both children and grandchildren, and having neither children nor grandchildren. We assessed whether the participants had any living children and grandchildren at the calendar year of their death. Only children and grandchildren who were alive at the time of the study were included in the analysis, since the number of participants who had lost a child was minimal.

Income variables were derived from the Norwegian Social Security Database. Income level was derived from the total annual income, including income from work, capital income, taxable transfers, and tax-free transfers. Income quartiles were created stratified by age and year of birth to account for age-related income variations and broader trends in income progression over time.

Source of income refers to the origin of an individual’s income and was classified into five categories. Working income includes individuals whose income is solely derived from employment, while retirement pension applies to individuals receiving only a retirement pension. Disability pension refers to individuals who receive only a disability pension. The mixed income category encompasses individuals with income from multiple sources, such as those transitioning from employment to retirement within the same year. No income includes individuals without registered income. For individuals who died by suicide, income level and source of income from the year prior to death were used, while income level and source of income from the year they were selected as controls were used for the controls.

Living arrangements were classified based on whether participants co-resided with others, and data from the Norwegian National Population Registry were used to identify living arrangements. Three categories were created: co-residential arrangement for individuals living with others, single residence arrangement for those living alone, and alternate living arrangement for individuals not registered with a private residence.

### Statistical analysis

We performed descriptive statistics for the cases and controls that are presented in cross-tables with frequencies and proportions. Chi-square tests were performed to explore the associations between sociodemographic factors and suicide death compared to the control group. The cross-tables and chi-square tests were stratified by sex. We then analyzed the statistical associations between sociodemographic factors and death by suicide by using conditional multivariate logistic regression analyses. This approach estimates the odds of the outcome, risk of suicide for each independent variable while controlling for the effects of all other included variables, thereby highlighting the independent contribution of each factor to the outcome. Death by suicide (binary yes/no) was the outcome variable, and educational level, urbanization level, marital status, family status, income level, and type of living arrangement were included as categorical independent variables. In addition, source of income was included in the analyses for the category 60 to 69 years of age. Since the retirement age in Norway is 67, and one cannot receive a disability pension past this age, the variable is not relevant for those 70 and older. The logistic regression analyses were stratified by sex (males and females) and age category (60 to 69, 70 to 79, and 80 and older). Results from the conditional multivariate logistic regression analyses are presented with odds ratios (ORs), p-values, and 95% confidence intervals (CIs). To address potential overlap and collinearity, we assessed pairwise associations between independent variables using Cramér’s V, stratified by sex. In addition, we conducted univariate conditional logistic regression analyses, stratified by sex and age category.

To counter the risk of Type I errors due to multiple testing, the predefined significance level of 0.05 was adjusted [15]. The Bonferroni correction was applied, adjusting the p-values based on the number of independent chi-square tests (eight, resulting in *p* = 0.05/8 = 0.006) and the number of independent variables included in the conditional multivariable logistic regression models (seven for the aged 60 to 69 group, *p* = 0.05/7 = 0.007; six for the 70 to 79, and 80 + groups, *p* = 0.05/6 = 0.008). Statistical analyses and figures were conducted using STATA 17 [16].

## Results

### Distribution of the dataset

Between 2005 and 2019, 2114 suicides were registered among individuals aged 60 and older in Norway. After excluding a total of 54 individuals, 2060 suicide cases were included in the analyses, of which 70.7% were males. The proportion of males among cases increased with age, comprising 68.1% of those aged 60 to 69, 72.2% of those aged 70 to 79, and 75.5% of those aged 80 and older.

The characteristics of individuals who died by suicide differed systematically from the control group when stratified by sex, as described in Table 1. Compared to the control groups, both males and females who died by suicide were more likely never to be married, divorced or separated, not have children and grandchildren, to have a lower income level, rely on a disability pension, and live in a single residence. Male suicide decedents were more likely to have no/primary education and have a lower income level, and female suicide decedents were more likely to live in urban areas.

**Table 1:** Descriptive statistics of the entire sample 60 years of age and older, stratified by sex

	Males						Females					
	Case		Control		Chi-Square test		Case		Control		Chi Square test	
	n	%	n	%	χ2	p-value	n	%	n	%	χ2	p-value
**Total**	1456		7280				604		3020			
**Age category**												
60–69 years	735	50.5	3675	50.5			345	57.1	1725	57.1		
70–79 years	419	28.8	2095	28.8			161	26.7	805	26.7		
80 years and older	302	20.7	1510	20.7			98	16.2	490	16.2		
**Educational level **					41.85	**<0.001**					1.08	0.583
No/primary education	505	34.7	1998	27.4			178	29.5	953	31.6		
Secondary education	698	47.9	3591	49.3			298	49.3	1458	48.3		
Higher education	253	17.4	1695	23.3			128	21.2	609	20.2		
**Immigration status **					4.43	0.035					0.06	0.803
Majority	1390	95.5	6848	94.1			569	94.2	2837	93.9		
Immigrated	66	4.5	432	5.9			35	5.8	183	6.1		
**Urbanization level**					1.51	0.47					48.23	**<0.001**
Urban	547	37.6	2680	36.8			316	52.3	1156	38.3		
Suburban	623	42.8	3066	42.1			226	37.4	1307	43.3		
Rural	286	19.6	1534	21.1			62	10.3	557	18.4		
**Marital status**					324.80	**<0.001**					129.83	**<0.001**
Never married	274	18.8	635	8.7			55	9.1	188	6.2		
Married	628	43.1	4902	67.3			202	33.4	1658	54.9		
Divorced/separated	353	24.2	1062	14.6			201	33.3	487	16.1		
Widowed	201	13.8	681	9.4			146	24.2	687	22.8		
**Family status**					123.95	**<0.001**					29.36	**<0.001**
Children	149	10.2	855	11.7			69	11.42	287	9.5		
Children and grandchildren	950	65.3	5471	75.2			424	70.02	2399	79.4		
No children or grandchildren	357	24.5	954	13.1			111	18.38	334	11.1		
**Income level**					87.16	**<0.001**					12.86	**0.005**
1 st quartile	238	16.4	797	11.0			259	42.9	1103	36.5		
2nd quartile	419	28.8	1683	23.1			162	26.8	849	28.1		
3rd quartile	424	29.1	2134	29.3			99	16.4	659	21.8		
4th quartile	375	25.8	2666	36.6			84	13.9	409	13.5		
**Source of income **					97.18	**<0.001**					87.71	**<0.001**
Working income	176	12.1	1194	16.4			40	6.6	442	14.6		
Retirement pension	701	48.3	3513	48.7			277	45.9	1466	48.5		
Disability pension	117	8.0	215	3.0			77	12.8	136	4.5		
Mixed income	443	30.4	2270	31.2			203	33.6	911	30.2		
No income	19	1.3	88	1.2			7	1.2	65	2.2		
**Living arrangement type**					400.04	**<0.001**					116.51	**< 0.001**
Single residence	700	48.1	1648	22.6			346	57.3	1026	34.0		
Co-residential arrangement	737	50.6	5516	75.8			251	41.6	1926	63.8		
Alternate living arrangement	19	1.3	116	1.6			7	1.2	68	2.3		

### Results from the conditional multivariable logistic regression analyses

**Fig. 1:**
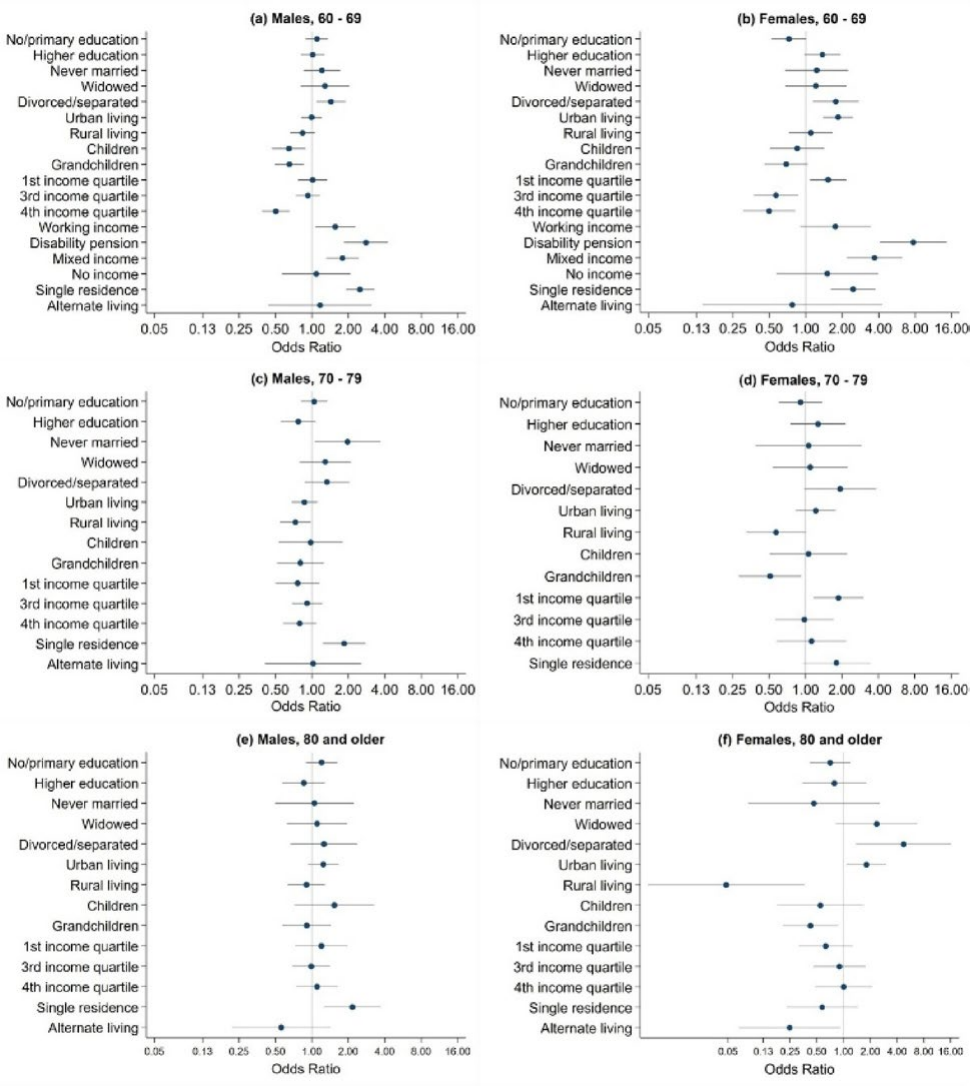
Conditional multivariable logistic regression analyses results by sex and age category

Table 2; Fig. 1 (a) and (b) presents the results from the multivariable logistic regression for the age category 60 to 69, stratified by sex. For both sexes, there was a significant increase in suicide risk when a disability pension (OR = 2.79 males, OR = 7.71 females) or mixed income (OR = 1.79 males, OR = 3.70 females) was the source of income compared to retirement pension. Living in a single residence, in contrast to co-residential arrangements, was also associated with an increased risk of suicide (OR = 2.49 males, OR = 2.46 females). For both males and females, there was a reduced risk for individuals in the fourth income level (OR = 0.50 males, OR = 0.50 females) compared to the second income level, and a reduced risk in males having children (OR = 0.64) and grandchildren (OR = 0.65). For females, results showed an increased risk of suicide in individuals living in urban areas (OR = 1.85) compared to suburban areas.

**Table 2:** Conditional multivariate logistic regression for the age category 60 to 69, stratified by sex

	Males			Females		
	OR	95% CI	p-value	OR	95% CI	p-value
**Educational level **						
No/primary education	1.100	0.89–1.36	0.369	0.725	0.52–1.01	0.055
Secondary education	1 (ref)	-	-	1 (ref)	-	-
Higher education	1.016	0.81–1.28	0.893	1.375	0.98–1.94	0.070
**Urbanization level**						
Urban	0.993	0.82–1.20	0.914	1.850	1.39–2.46	**<0.001**
Suburban	1 (ref)	-	-	1 (ref)	-	-
Rural	0.836	0.66–1.06	0.134	1.101	0.73–1.66	0.650
**Marital status**						
Never married	1.211	0.85–1.72	0.282	1.231	0.67–2.25	0.500
Married	1 (ref)	-	-	1 (ref)	-	-
Divorced/separated	1.438	1.09–1.90	0.010	1.769	1.15–2.73	0.010
Widowed	1.281	0.81–2.03	0.292	1.211	0.68–2.16	0.518
**Family status**						
Children	0.644	0.47–0.89	**0.007**	0.850	0.51–1.42	0.535
Children and grandchildren	0.651	0.50–0.85	**0.002**	0.689	0.46–1.04	0.076
No children or grandchildren	1(ref)	-	-	1 (ref)	-	-
**Income level**						
1^st^ quartile	1.014	0.77–1.33	0.923	1.529	1.08–2.16	0.016
2^nd^ quartile	1 (ref)	-	-	1 (ref)	-	-
3^rd^ quartile	0.928	0.74–1.17	0.525	0.567	0.37–0.87	0.008
4^th^ quartile	0.503	0.39–0.65	**<0.001**	0.498	0.30–0.82	**0.006**
**Source of income **						
Working income	1.556	1.07–2.27	0.021	1.755	0.90–3.42	0.099
Retirement pension	1 (ref)	-	-	1 (ref)	-	-
Disability pension	2.795	1.84–4.24	**<0.001**	7.721	4.09–14.57	**<0.001**
Mixed income	1.788	1.32–2.43	**<0.001**	3.702	1.69–3.90	**<0.001**
No income	1.085	0.57–2.08	0.805	1.510	0.58–3.96	0.402
**Living arrangement type**						
Single residence	2.491	1.91–3.26	**<0.001**	2.461	1.61–3.77	**<0.001**
Co-residential arrangement	1 (ref)	-	-	1 (ref)	-	-
Alternate living arrangement	1.167	0.44–3.12	0.759	0.774	0.14–4.27	0.768

Abbreviations: OR = Odds ratios, CI = Confidence interval, ref = Reference category

*Significance level <0.007, significant p-values are in bold

Table 3; Fig. 1 (c) and (d) shows the results from the multiple logistic regression for the age category 70 to 79, stratified by sex. For males, there was a significant increase in the suicide risk among those living in a single residence (OR = 1.85) compared to a co-residential arrangement.

**Table 3:** Conditional multivariate logistic regression for the age category 70 to 79, stratified by sex

	Males			Females		
	OR	95% CI	p-value	OR	95% CI	p-value
**Educational level **						
No/primary education	1.044	0.81–1.34	0.733	0.911	0.60–1.38	0.659
Secondary education	1 (ref)	-	-	1 (ref)	-	-
Higher education	0.771	0.55–1.08	0.127	1.271	0.75–2.15	0.369
**Urbanization level**						
Urban	0.867	0.68–1.11	0.262	1.216	0.83–1.78	0.313
Suburban	1 (ref)	-	-	1 (ref)	-	-
Rural	0.731	0.55–0.98	0.037	0.571	0.33–1.01	0.053
**Marital status**						
Never married	1.966	1.05–3.67	0.033	1.062	0.39–2.92	0.908
Married	1 (ref)	-	-	1 (ref)	-	-
Divorced/separated	1.333	0.87–2.04	0.183	1.934	0.97–3.84	0.060
Widowed	1.288	0.79–2.10	0.309	1.094	0.54–2.24	0.805
**Family status**						
Children	0.976	0.53–1.80	0.262	1.058	0.51–2.22	0.881
Children and grandchildren	0.803	0.55–1.15	0.037	0.510	0.28–0.92	0.026
No children or grandchildren	1(ref)	-	-	1 (ref)	-	-
**Income level**						
1^st^ quartile	0.761	0.50–1.16	0.200	1.874	1.17–3.01	0.009
2^nd^ quartile	1 (ref)	-	-	1 (ref)	-	-
3^rd^ quartile	0.912	0.69–1.21	0.527	0.981	0.57–1.70	0.944
4^th^ quartile	0.793	0.58–1.09	0.150	1.121	0.58–2.16	0.732
**Living arrangement type**						
Single residence	1.847	1.23–2.78	**0.003**	1.802	0.95–3.41	0.071
Co-residential arrangement	1 (ref)	-	-	1 (ref)	-	-
Alternate living arrangement	1.024	0.41–2.55	0.959	-	Empty	-

Abbreviations: OR = Odds ratios, CI = Confidence interval, ref = Reference category

*Significance level <0.008, significant p-values are in bold

Table 4; Fig. 1 (e) and (f) shows the results from the multiple logistic regression model for the age category 80 and older, stratified by sex. For males, there was a significant increase in suicide risk among those living in a single residence (OR = 2.16) compared to co-residential arrangement. For females, there is a significantly reduced suicide risk in those living in rural areas (OR = 0.05), compared to suburban areas.

**Table 4:** Conditional multivariate logistic regression for the age category 80 and older, stratified by sex

	Males			Females		
	OR	95% CI	p-value	OR	95% CI	p-value
**Educational level **						
No/primary education	1.202	0.89–1.61	0.219	0.706	0.42–1.20	0.196
Secondary education	1 (ref)	-	-	1 (ref)	-	-
Higher education	0.858	0.57–1.28	0.457	0.571	0.34–1.80	0.571
**Urbanization level**						
Urban	1.243	0.93–1.67	0.146	1.804	1.08–3.01	0.024
Suburban	1 (ref)	-	-	1 (ref)	-	-
Rural	0.902	0.63–1.29	0.568	0.048	0.01–0.36	**0.003**
**Marital status**						
Never married	1.049	0.50–2.21	0.900	0.463	0.08–2.55	0.376
Married	1 (ref)	-	-	1 (ref)	-	-
Divorced/separated	1.258	0.67–2.37	0.479	4.725	1.38–16.23	0.014
Widowed	1.104	0.62–1.96	0.736	2.356	0.82–6.77	0.111
**Family status**						
Children	1.531	0.72–3.25	0.267	0.548	0.18–1.67	0.209
Children and grandchildren	0.908	0.57–1.44	0.679	0.424	0.21–0.87	0.020
No children or grandchildren	1(ref)	-	-	1 (ref)	-	-
**Income level**						
1^st^ quartile	1.198	0.73–1.97	0.475	0.629	0.32–1.26	0.189
2^nd^ quartile	1 (ref)	-	-	1 (ref)	-	-
3^rd^ quartile	0.987	0.69–1.41	0.941	0.900	0.46–1.75	0.748
4^th^ quartile	1.103	0.75–1.63	0.624	1.002	0.48–2.10	0.996
**Living arrangement type**						
Single residence	2.162	1.26–3.71	**0.005**	0.574	0.23–1.45	0.240
Co-residential arrangement	1 (ref)	-	-	1 (ref)	-	-
Alternate living arrangement	0.559	0.22–1.43	0.223	0.247	0.07–0.92	0.037

Abbreviations: OR = Odds ratios, CI = Confidence interval, ref = Reference category

*Significance level <0.008, significant p-values are in bold

Collinearity analyses indicated a strong association between marital status and living arrangement (Cramér’s V = 0.56 males, 0.58 females). Moderate associations were observed between marital status and family status (Cramér’s V = 0.46 males, 0.40 females) and between educational level and family status (Cramér’s V = 0.30 males, 0.31 females). All other associations were < 0.3. Detailed results are available in supplementary Table S1. Results from the univariate conditional logistic regression analyses, stratified by sex and age category, are presented in supplementary Table S2 (60–69 years), Table S3 (70–79 years), and Table S4 (80 and older).

## **Discussion**

This study is the first comprehensive examination of sociodemographic risk factors and death by suicide among older adults in Norway, utilizing linked nation-wide registries. The main findings are: 1) for males across all old age categories, living alone was consistently associated with an increased suicide risk; 2) for females aged 60 to 69, living in urban areas was associated with increased suicide risk, while for females 80 and older living in rural areas was a protective factor; and 3) across both sexes between 60 to 69 years of age, relying on a disability pension or a mixed income significantly increased suicide risk.

### **Living arrangements and suicide risk **

Living alone was significantly associated with increased suicide risk among males across all age categories, while for females, this association was only found in the 60 to 69 age category. A systematic review [17] found mixed results, but the present results are supported by a recent population-based study from United Kingdom showing increased risk among people 55 or older who lived alone [4]. 

The differences between sexes among the older age groups in the present study may be explained by differences in help-seeking behavior. One study report that, compared to females, males living alone experience lower help- seeking intension [18]. Another recent study [19] found that being both older and male characterized those who died by suicide without seeking help. 

Social support is a well-established protective factor against suicide, and individuals who live alone may lack the social engagement and emotional support necessary to cope with life’s challenges [20]. Living alone increases the risk of loneliness, and loneliness and lack of social engagement are associated with suicidal behavior [17, 21]. A recent Norwegian study found that among individuals living alone, 60% of men and 51% of women reported experiencing loneliness to some degree. The highest proportion of loneliness was reported by those aged 80 years and older [22]. At the same time, studies indicate that living alone poses a greater all-cause mortality risk for males than for females, particularly concerning social isolation and associated health outcomes [23, 24]. The association could be partly explained by unmeasured health conditions, which we did not include in the analyses. 

Differences in suicide risk among the oldest, between males and females, may be attributed to traditional gender roles and socialization patterns. Females often maintain broader and more diverse social networks, engaging in activities that foster social connections beyond the closest family [25, 26]. In contrast, males have historically relied more on their spouses for emotional support and social engagement [27]. Consequently, the loss of a partner can lead to a more significant disruption in males’ social networks, increasing their vulnerability to social isolation [28]. 

Although marital status was not independently associated with suicide in the logistic regression analyses, its potential role should be addressed. We found a strong statistical association between marital status and living arrangement, which may suggest that marital status indirectly influences suicide risk through its impact on living arrangements.

### **Urbanization level and suicide risk **

The present study found that living in urban areas was associated with a higher suicide risk for females aged 60 to 69, compared to those living in suburban areas. This result is consistent with a study from Italy that demonstrated that older women in urban environments may face unique challenges that increase their vulnerability to suicide [5]. Urban areas, while providing better access to healthcare and social services, can also foster greater social isolation, anonymity, and mental health stressors due to the fast-paced and crowded nature of city life [29]. Why females living in urban areas are more vulnerable to and have higher suicide risks than males remains unclear. Urban living for older adults, especially for females, may be associated with higher level of loneliness and mental health problems, which in turn can increase suicide risk [30, 31].The increased pace of life and the difficulty of navigating large urban environments might create emotional and physical burdens for older individuals. As health-related data were not included, we cannot rule out that underlying health differences between urban and rural populations contributed to these patterns.

### **Income source and suicide risk **

Our findings indicate that receiving a disability pension was associated with a higher suicide risk in the youngest group of older adults between 60 and 69, with the highest risk (OR) being among females. This aligns with previous research, which has identified that individuals on disability pensions have significantly increased suicide risk due to financial strain and underlying health conditions [32]. Both physical and mental health illness can increase the risk of suicide and be the cause of receiving a disability pension. Individuals with chronic health conditions, who often rely on disability benefits, might be at increased risk of suicide, partly due to the stress of managing both health problems and financial insecurity. As we did not include health data in our analyses, the observed association between disability pension and suicide risk may be influenced by unmeasured confounding from underlying health conditions. 

In addition, our study found increased suicide risk in the mixed income group compared to the retired group. The mixed income group include those receiving income from more than one source within the given year, when transitioning from a disability pension to retirement pension or from working income to retirement pension. It is possible that the significant finding is due to the group that transitioned from a disability pension to retirement pension, however, this should be investigated further, since previous research has shown that transition to retirement might increase risk, especially for those who retire involuntarily [9]. 

### **Income level and suicide risk**

For both males and females in the 60 to 69 age group, our study show that higher income levels were associated with a reduced suicide risk, which is consistent with previous findings from a Swedish study that showed that economic factors influenced risk for the youngest of the old, but not in the oldest of the old [21]. Choi et al. [33] reported an increased risk in those aged 64 to 75 with a lower income, but not in the 75+ age group. Older adults with higher income tend to experience fewer functional limitations [34], while those with lower income are more likely to experience social isolation [35]. Educational attainment and labor market attachment influence both physical and mental health conditions and, conversely, health status can affect these socioeconomic factors, collectively contributing to suicide risk. [36]. While income may capture important social and economic resources, unmeasured health factors could account for part of the observed association.

### **Strength and limitations **

A strength of the study is the use of high-quality register data including all registered suicides in the observation period and using a case-control design. Because the use of secondary data is regulated by national laws and registration is mandatory, the amount of missing data in our study is low. Specifically, only 1.8% of cases were excluded due to missing independent variables, underscoring the robustness of the dataset. Although the proportion of missing data and exclusions was low (2.5%), the observed differences in sex and immigration status indicate that some degree of selection bias cannot be fully ruled out. The data from health registers, including the quality of registered causes of death, specifically suicide, is of high quality [37]. Statistically, suicide is a rare event, making register data an appropriate data source to investigate the characteristics of suicide decedents. Having access to registry data, which is representative for the entire population, gives a unique opportunity to study sociodemographic factors across sex and age. 

The study has several limitations. We relied on secondary data, primarily collected for administrative purposes rather than research. There are limitations in the data available: for instance, marital status only includes “married” people and not cohabitants. For some subgroup analyses, particularly among the oldest females, statistical power may have been limited; although effect sizes suggested potential associations, the lack of statistical significance could reflect insufficient power rather than the absence of an effect. The strong and moderate associations between some independent variables underscore the need for caution in interpretation, as correlated factors may limit the ability to distinguish their independent effects. 

Furthermore, we utilized cross-sectional measurements, which limits the ability to draw causal conclusions. Although the study matched and adjusted for key variables, relevant variables such as the quality of social support, loneliness, social isolation, and social support and are not available from the registers. Moreover, the study did not include detailed measures of support services or somatic and mental health, all of which are known to affect suicidal behavior [38]. These should be included in future studies to explore the associations further. 

### **Implications and future research **

The increased vulnerability of individuals living alone suggests that interventions aimed at improving social connectedness, such as community-based programs and social networks, could help decrease suicide risk, and improve quality of life and mental health [39]. Outreach programs targeting those living alone, particularly in the older male population, may be effective in reducing isolation and providing support. Future research should further explore the interplay between living arrangements, social support, and psychological well-being to better identify individuals at risk.

 For females, living in urban areas was associated with an increased risk of suicide compared to those living in suburban areas. These findings have important implications for urban-focused suicide prevention programs. Tailored interventions that emphasize community integration, accessible mental health care, and support services could help decrease suicide risk. In addition, more research is needed on the urban-rural divide in suicide risk, particularly for older females. While urban living has been associated with increased suicide risk in this study, it remains unclear what specific urban characteristics contribute to this effect. Studies examining the role of social support networks, access to mental health services, and socioeconomic challenges in urban environments will provide a clearer picture of the mechanisms behind this increased risk. 

Our study found that disability pension recipients have a significantly higher suicide risk, among both sexes aged 60 to 69. Disability pensions are often associated with chronic health conditions or disability, both of which can lead to social isolation and economic hardship. These factors are crucial in understanding the increased suicide risk for individuals receiving disability benefits. It is important to note that health and economic vulnerabilities are likely intertwined for these individuals, meaning that interventions should not only focus on financial stability but also address the physical and mental health challenges faced by this group. Future research should further explore the relationship between disability pensions and mental health in older adults. Investigating the causal relationships between these factors and mental health outcomes will be important for developing targeted interventions.

 In addition, our analyses suggests that the presence of children and grandchildren may act as a protective factor for males between the ages of 60 and 69. To better understand this relationship, future research should incorporate variables related to frequency and quality of family interactions, the availability of both informal and formal caregiving, and perceived social support. A longitudinal approach is recommended to capture temporal changes, with particular attention given to variations across sex and age groups. This study highlights the importance of sex differences in suicide risk for older adults. Our findings emphasize the critical role of sex differences in suicide risk in later life. Integrating a sex-specific perspective into future research and preventive interventions is essential to ensure more effective and targeted outcomes.

## **Conclusion**

Living arrangements, urbanization level, and source of income are important risk factors for suicide among older adults in Norway. Community-focused initiatives aimed at fostering social connectedness, while addressing differences linked to urban-rural divides, sex, and age, might be useful initiatives, enhance mental health, and improve the overall quality of life for older adults. 

## Supplementary Information

Below is the link to the electronic supplementary material.

Supplementary material

## Data Availability

The data used in this study were obtained from a third party and are not publicly available. As part of a project funded by the Research Council of Norway (project number 288731), the researchers received de-identified data files from the registry holders. Access to the data can be applied for from the registry holders, subject to legal and ethical approval.
