# Supplementary material for: Suicide and sociodemographic factors among older adults in Norway: a register-based study
Source: Soc Psychiatry Psychiatr Epidemiol. 2025 Oct 21;61(3):505–16. doi: 10.1007/s00127-025-03007-x (PMC12995991; doi:10.1007/s00127-025-03007-x)
Supplement: Supplementary file 1 — Supplementary material [file 127_2025_3007_MOESM1_ESM.docx]

**Title**

Suicide and Sociodemographic Factors Among Older Adults in Norway: A Register-Based Study

**Journal name**

Social Psychiatry and Psychiatric Epidemiology

**Author information**

**Author names**

Anna Torp Johansen^1,2^, Sissel Marguerite Bélanger^3^, Anne Reneflot^3^, Erlend Hem^2,4^, Eivind Aakhus^5^, Carine Øien-Ødegaard^3^, Kim Stene-Larsen^3^, Cecilie Bhandari Hartberg^6^

**Affiliations**

^1^ Department of Old Age Psychiatry, Division of Mental Health and Addiction, Oslo University Hospital, Oslo, Norway

^2^ Department of Behavioral Science in Medicine, Institute of Basic Medical Sciences, Faculty of Medicine, University of Oslo, Norway

^3^ Department of Mental Health, Norwegian Institute of Public Health, Oslo, Norway

^4^ Institute for Studies of the Medical Profession, Oslo, Norway

^5^ National Norwegian Centre for Ageing and Health, Vestfold Hospital Trust, Tønsberg, Norway

^6^ Division of Mental Health and Addiction, Oslo University Hospital, Oslo, Norway

**Corresponding author**

Name: Anna Torp Johansen, MD

E-mail: [a.t.johansen@ous-research.no](mailto:a.t.johansen@ous-research.no)


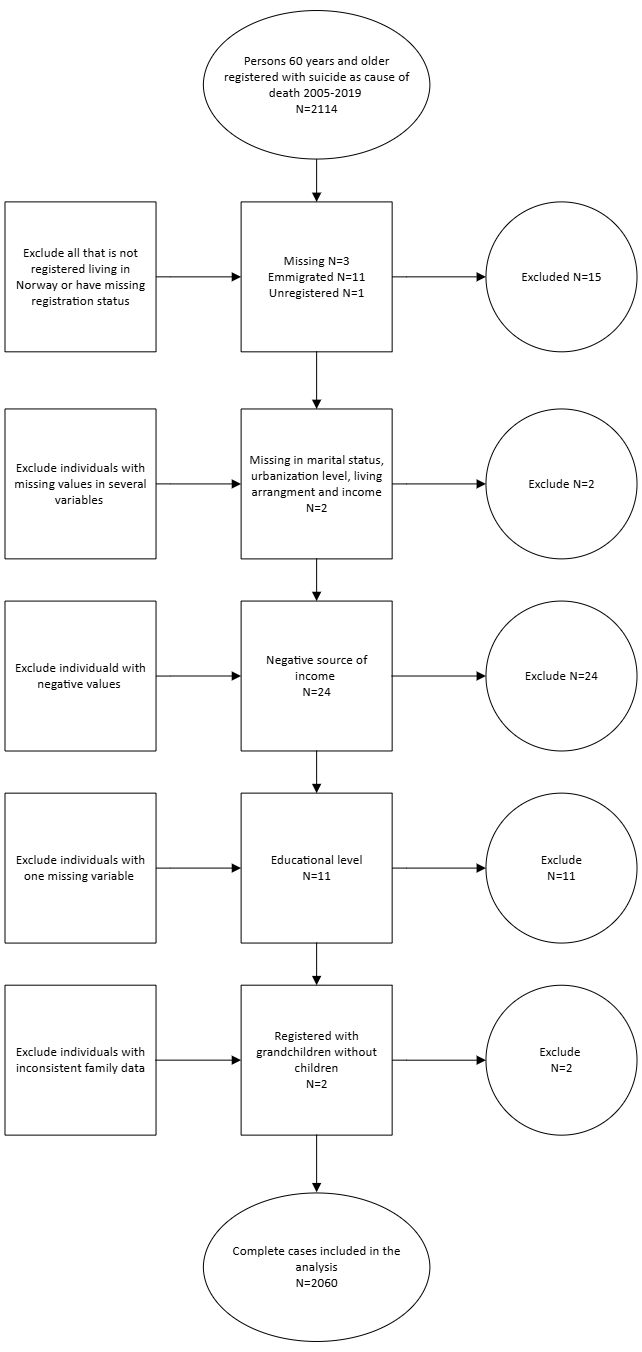


**Figure S1** Flow chart illustrating the exclusion process of potential cases

**Table S1** Strength of association between independent variables, Cramér’s V stratified by sex

| **Independent variables** | **Males** | **Females** |
| --- | --- | --- |
|  | **Cramér’s V** | **Cramér’s V** |
| Education level vs. Urbanization level | 0.13 | 0.11 |
| Education level vs. Marital status | 0.11 | 0.13 |
| Education level vs. Family status | 0.11 | 0.10 |
| Education level vs. Income level | **0.30** | **0.31** |
| Education level vs. Source of income | 0.16 | 0.19 |
| Education level vs. Living arrangement type | 0.09 | 0.05 |
| Urbanization level vs. Marital status | 0.06 | 0.06 |
| Urbanization level vs. Family status | 0.06 | 0.07 |
| Urbanization level vs. Income level | 0.14 | 0.14 |
| Urbanization level vs. Source of income | 0.04 | 0.05 |
| Urbanization level vs. Living arrangement type | 0.04 | 0.04 |
| Marital status vs. Family status | **0.46** | **0.40** |
| Marital status vs. Income level | 0.15 | 0.13 |
| Marital status vs. Source of income | 0.17 | 0.20 |
| Marital status vs. Living arrangement type | **0.56** | **0.58** |
| Family status vs. Income level | 0.15 | 0.09 |
| Family status vs. Source of income | 0.19 | 0.11 |
| Family status vs. Living arrangement type | 0.23 | 0.14 |
| Income level vs. Source of income | 0.26 | 0.23 |
| Income level vs. Living arrangement type | 0.12 | 0.14 |
| Source of income vs. Living arrangement type | 0.13 | 0.17 |

Moderate associations are in bold

Strong associations are in bold and underlined

**Table S2** Conditional univariate logistic regression for the age category 60 to 69, stratified by sex

|  | **Males** | |  | |  | | **Females** | |  | |  |
| --- | --- | --- | --- | --- | --- | --- | --- | --- | --- | --- | --- |
|  | **OR** | **95% CI** | | **p-value** | | **OR** | | **95% CI** | | **p-value** | |
| **Educational level** |  |  | |  | |  | |  | |  | |
| No/primary education | 1.474 | 1.22-1.78 | | **<0.001** | | 1.047 | | 0.79-1.39 | | 0.747 | |
| Secondary education | 1 (ref) | - | | - | | 1 (ref) | | - | | - | |
| Higher education | 0.773 | 0.63-0.95 | | 0.015 | | 0.981 | | 0.74-1.30 | | 0.896 | |
| **Urbanization level** |  |  | |  | |  | |  | |  | |
| Urban | 1.016 | 0.84-1.22 | | 0.855 | | 1.641 | | 1.28-2.11 | | **<0.001** | |
| Suburban | 1 (ref) | - | | - | | 1 (ref) | | - | | - | |
| Rural | 0.954 | 0.77-1.19 | | 0.671 | | 0.960 | | 0.66-1.39 | | 0.829 | |
| **Marital status** |  |  | |  | |  | |  | |  | |
| Never married | 3.928 | 3.14-4.91 | | **<0.001** | | 3.027 | | 2.02-4.53 | | **<0.001** | |
| Married | 1 (ref) | - | | - | | 1 (ref) | | - | | - | |
| Divorced/separated | 3.031 | 2.50-3.68 | | **<0.001** | | 3.746 | | 2.85-4.93 | | **0.001** | |
| Widowed | 2.461 | 1.65-3.68 | | **<0.001** | | 2.046 | | 1.36-3.07 | | **<0.001** | |
| **Family status** |  |  | |  | |  | |  | |  | |
| Children | 0.379 | 0.29-0.49 | | **<0.001** | | 0.596 | | 0.39-1.92 | | 0.018 | |
| Children and grandchildren | 0.400 | 0.33-0.48 | | **<0.001** | | 0.528 | | 0.39-0.72 | | **<0.001** | |
| No children or grandchildren | 1(ref) | - | | - | | 1 (ref) | | - | | - | |
| **Income level** |  |  | |  | |  | |  | |  | |
| 1^st^ quartile | 1.326 | 1.05-1.67 | | 0.017 | | 1.358 | | 1.03-1.79 | | 0.031 | |
| 2^nd^ quartile | 1 (ref) | - | | - | | 1 (ref) | | - | | - | |
| 3^rd^ quartile | 0.729 | 0.59-0.90 | | **0.003** | | 0.568 | | 0.39-0.83 | | **0.004** | |
| 4^th^ quartile | 0.400 | 0.32-0.50 | | **<0.001** | | 0.728 | | 0.48-1.11 | | 0.141 | |
| **Source of income** |  |  | |  | |  | |  | |  | |
| Working income | 1.029 | 0.73-1.46 | | 0.871 | | 1.166 | | 0.64-2.11 | | 0.613 | |
| Retirement pension | 1 (ref) | - | | - | | 1 (ref) | | - | | - | |
| Disability pension | 3.914 | 2.69-5.69 | | **<0.001** | | 7.598 | | 4.32-13.37 | | **<0.001** | |
| Mixed income | 1.450 | 1.09-1.94 | | 0.012 | | 2.991 | | 1.86-4.80 | | **<0.001** | |
| No income | 1.520 | 0.83-2.77 | | 0.171 | | 1.488 | | 0.60-3.72 | | 0.395 | |
| **Living arrangement type** |  |  | |  | |  | |  | |  | |
| Single residence | 4.015 | 3.38-4.76 | | **<0.001** | | 3.608 | | 2.83-4.61 | | **<0.001** | |
| Co-residential arrangement | 1 (ref) | - | | - | | 1 (ref) | | - | | - | |
| Alternate living arrangement | 2.438 | 0.96-6.17 | | 0.060 | | 2.280 | | 0.47-11.16 | | 0.309 | |

Abbreviations: OR = Odds ratios, CI = Confidence interval, ref = Reference category

*Significance level <0.007, significant p-values are in bold

**Table S3** Conditional univariate logistic regression for the age category 70 to 79, stratified by sex

|  | **Males** | | | | **Females** | | |
| --- | --- | --- | --- | --- | --- | --- | --- |
|  | **OR** | **95% CI** | **p-value** | **OR** | | **95% CI** | **p-value** |
| **Educational level** |  |  |  |  | |  |  |
| No/primary education | 1.216 | 0.96-1.54 | 0.100 | 0.966 | | 0.66-1.42 | 0.860 |
| Secondary education | 1 (ref) | - | - | 1 (ref) | | - | - |
| Higher education | 0.673 | 0.49-0.91 | 0.012 | 1.324 | | 0.83-2.07 | 0.240 |
| **Urbanization level** |  |  |  |  | |  |  |
| Urban | 0.860 | 0.68-1.09 | 0.209 | 1.244 | | 0.87-1.78 | 0.229 |
| Suburban | 1 (ref) | - | - | 1 (ref) | | - | - |
| Rural | 0.849 | 0.64-1.12 | 0.251 | 0.599 | | 0.35-1.04 | 0.068 |
| **Marital status** |  |  |  |  | |  |  |
| Never married | 3.933 | 2.81-5.50 | **<0.001** | 2.504 | | 1.23-5.09 | 0.908 |
| Married | 1 (ref) | - | - | 1 (ref) | | - | - |
| Divorced/separated | 2.145 | 1.62-2.85 | **<0.001** | 2.760 | | 1.79-4.26 | **<0.001** |
| Widowed | 2.249 | 1.61-3.14 | **<0.001** | 1.566 | | 0.01-2.42 | 0.043 |
| **Family status** |  |  |  |  | |  |  |
| Children | 0.538 | 0.33-0.89 | 0.016 | 1.0136 | | 0.58-2.24 | 0.713 |
| Children and grandchildren | 0.439 | 0.33-0.58 | **<0.001** | 0.503 | | 0.31-0.82 | **0.005** |
| No children or grandchildren | 1(ref) | - | - | 1 (ref) | | - | - |
| **Income level** |  |  |  |  | |  |  |
| 1^st^ quartile | 0.861 | 0.58-1.28 | 0.460 | 1.427 | | 0.92-2.21 | 0.110 |
| 2^nd^ quartile | 1 (ref) | - | - | 1 (ref) | | - | - |
| 3^rd^ quartile | 0.800 | 0.61-1.05 | 0.104 | 1.141 | | 0.68-1.92 | 0.619 |
| 4^th^ quartile | 0.601 | 0.46-0.79 | **<0.001** | 1.620 | | 0.91-2.88 | 0.101 |
| **Living arrangement type** |  |  |  |  | |  |  |
| Single residence | 2.749 | 2.20-3.44 | **<0.001** | 2.182 | | 1.54-3.09 | **<0.001** |
| Co-residential arrangement | 1 (ref) | - | - | 1 (ref) | | - | - |
| Alternate living arrangement | 1.683 | 0.73-3.90 | 0.225 | - | | Empty | - |

Abbreviations: OR = Odds ratios, CI = Confidence interval, ref = Reference category

*Significance level <0.008, significant p-values are in bold

**Table S4** Conditional univariate logistic regression for the age category 80 and older, stratified by sex

|  | **Males** | | | | **Females** | | |
| --- | --- | --- | --- | --- | --- | --- | --- |
|  | **OR** | **95% CI** | **p-value** | **OR** | | **95% CI** | **p-value** |
| **Educational level** |  |  |  |  | |  |  |
| No/primary education | 1.189 | 0.91-1.56 | 0.210 | 0.592 | | 0.37-0.94 | 0.027 |
| Secondary education | 1 (ref) | - | - | 1 (ref) | | - | - |
| Higher education | 0.922 | 0.64-1.33 | 0.665 | 0.920 | | 0.46-1.86 | 0.816 |
| **Urbanization level** |  |  |  |  | |  |  |
| Urban | 1.202 | 0.91-1.58 | 0.188 | 2.001 | | 1.24-3.22 | **0.004** |
| Suburban | 1 (ref) | - | - | 1 (ref) | | - | - |
| Rural | 0.935 | 0.67-1.31 | 0.700 | 0.045 | | 0.01-0.34 | **0.002** |
| **Marital status** |  |  |  |  | |  |  |
| Never married | 2.146 | 0142-3.25 | **<0.001** | 0.560 | | 0.151-2.080 | 0.386 |
| Married | 1 (ref) | - | - | 1 (ref) | | - | - |
| Divorced/separated | 2.263 | 1.44-3.54 | **<0.001** | 3.583 | | 1.50-8.57 | **0.004** |
| Widowed | 2.181 | 1.62-2.93 | **<0.001** | 1.342 | | 0.74-2.44 | 0.337 |
| **Family status** |  |  |  |  | |  |  |
| Children | 1.224 | 0.63-2.37 | 0.548 | 1.062 | | 0.40-2.82 | 0.904 |
| Children and grandchildren | 0.701 | 0.51-0.97 | 0.030 | 0.611 | | 0.34-1.08 | 0.092 |
| No children or grandchildren | 1(ref) | - | - | 1 (ref) | | - | - |
| **Income level** |  |  |  |  | |  |  |
| 1^st^ quartile | 1.192 | 0.74-1.92 | 0.470 | 0.478 | | 0.26-0.90 | 0.021 |
| 2^nd^ quartile | 1 (ref) | - | - | 1 (ref) | | - | - |
| 3^rd^ quartile | 1.025 | 0.73-1.45 | 0.888 | 1.114 | | 0.61-2.03 | 0.725 |
| 4^th^ quartile | 1.037 | 0.75-1.44 | 0.831 | 1.554 | | 0.86-2.82 | 0.146 |
| **Living arrangement type** |  |  |  |  | |  |  |
| Single residence | 2.461 | 1.90-3.20 | **<0.001** | 1.349 | | 0.81-2.26 | 0.254 |
| Co-residential arrangement | 1 (ref) | - | - | 1 (ref) | | - | - |
| Alternate living arrangement | 0.653 | 0.28-1.54 | 0.332 | 0.540 | | 0.19-1.50 | 0.237 |

Abbreviations: OR = Odds ratios, CI = Confidence interval, ref = Reference category

*Significance level <0.008, significant p-values are in bold
